# A systematic review of predictive models for asthma development in children

**DOI:** 10.1186/s12911-015-0224-9

**Published:** 2015-11-28

**Authors:** Gang Luo, Flory L. Nkoy, Bryan L. Stone, Darell Schmick, Michael D. Johnson

**Affiliations:** 1 Department of Biomedical Informatics, University of Utah, Suite 140, 421 Wakara Way, Salt Lake City, UT 84108 USA; 2 Department of Pediatrics, University of Utah, 100 N Mario Capecchi Drive, Salt Lake City, UT 84113 USA; 3 Spencer S. Eccles Health Sciences Library, 10 N 1900 E, Salt Lake City, UT 84112 USA

**Keywords:** Asthma development, Bronchiolitis, Predictive model, Machine learning

## Abstract

**Background:**

Asthma is the most common pediatric chronic disease affecting 9.6 % of American children. Delay in asthma diagnosis is prevalent, resulting in suboptimal asthma management. To help avoid delay in asthma diagnosis and advance asthma prevention research, researchers have proposed various models to predict asthma development in children. This paper reviews these models.

**Methods:**

A systematic review was conducted through searching in PubMed, EMBASE, CINAHL, Scopus, the Cochrane Library, the ACM Digital Library, IEEE Xplore, and OpenGrey up to June 3, 2015. The literature on predictive models for asthma development in children was retrieved, with search results limited to human subjects and children (birth to 18 years). Two independent reviewers screened the literature, performed data extraction, and assessed article quality.

**Results:**

The literature search returned 13,101 references in total. After manual review, 32 of these references were determined to be relevant and are discussed in the paper. We identify several limitations of existing predictive models for asthma development in children, and provide preliminary thoughts on how to address these limitations.

**Conclusions:**

Existing predictive models for asthma development in children have inadequate accuracy. Efforts to improve these models’ performance are needed, but are limited by a lack of a gold standard for asthma development in children.

**Electronic supplementary material:**

The online version of this article (doi:10.1186/s12911-015-0224-9) contains supplementary material, which is available to authorized users.

## Background

Asthma is a chronic lung disease caused by airway inflammation. Asthma is the most common pediatric chronic disease [1, 2] affecting 7.1 million (9.6 %) of American children [3, 4]. Asthma is the primary diagnosis for 1/3 of pediatric emergency department visits [5], and the most frequent reason for preventable pediatric hospitalization [6] and school absenteeism due to chronic conditions [7]. In 2008, 9.3 billion dollars, or 8 % of the total direct healthcare cost for all children, were spent on pediatric asthma [1].

About 80 % of pediatric asthma patients have symptom onset before age six [8, 9], most of them before age three [10–12]. However, only about 1/3 of children with at least one episode of asthmatic symptoms by age three will have asthma at age six and over [10, 13–18]. Asthma is under-diagnosed in 18–75 % of asthmatic children [19–23]. Overdiagnosis of asthma is also prevalent. Eleven percent of patients in primary care using inhaled corticosteroids, the most potent and consistently effective long-term control medication for asthma [24, 25], have no indication for the medication [26]. It is desirable to construct an accurate model to predict whether a child will develop asthma in the future. In support of the potential of predictive models, a published predictive model for asthma development has already been shown to outperform a physician’s diagnosis of asthma in young children, which had a low sensitivity of 29 % and a low positive predictive value of 23 % [27]. Such a model can provide several benefits.

First, appropriate asthma treatment can prevent serious asthma complications. A delay (median = 3.3 years) in diagnosis is experienced by 2/3 of asthmatic children [28–34] and is associated with suboptimal or no treatment for asthma [19, 20, 28, 35, 36], presenting a major clinical and public health concern [29, 37]. Many children, including 37 % of the 32 million American children on Medicaid in 2013, miss regular check-ups [38]. By identifying children at high risk for asthma and scheduling more frequent follow-up with a clinician familiar with asthma, the clinician can diagnose asthma in a timely manner and start asthma treatment earlier [39]. This has long-term benefits including fewer respiratory symptoms [40–47], reduced maintenance dose of asthma control medication [43, 48], fewer medication side effects [24], less need for secondary medications [40, 42–44, 46–48], reduced overuse of antibiotics [29], fewer asthma exacerbations [31, 41–53], less school absenteeism [45, 47], fewer caregiver work days lost [53], lower healthcare costs [24, 43, 48, 50, 53], preserved lung function avoiding airway remodeling (i.e., permanent alterations in the airway structure) [31, 41, 43, 44, 47, 48, 52, 54–58], less need for rehabilitation [50], lower risk of death from asthma [31, 50, 59], increased chance that the patient outgrows his/her asthma [60], and improved quality of life [14, 54]. Moreover, timely asthma treatment can benefit both children with severe asthma and children with mild asthma [49, 61–63].

Second, asthma is a subjective, clinical diagnosis in children under five [14, 64, 65]. Clinicians have difficulty diagnosing asthma in young children [27, 64, 66]. Most children under five cannot cooperate reliably with objective lung function measurements. Also, there is no genetic marker or diagnostic test that can reliably diagnose asthma [64, 67, 68]. Using a predictive model can help physicians better diagnose asthma [69, 70], particularly in children under five.

Third, the information provided by a predictive model can contribute directly to children’s quality of life. A low predicted risk for asthma can alleviate concerns of the child and his/her caregivers [71]. A high predicted risk may help the child and caregivers understand symptoms, improve treatment adherence, and adjust lifestyle and living conditions to avoid exposing the child to environmental contaminants and allergens [45, 72].

Fourth, proposed preventive interventions for asthma [73–82] such as suplatast tosilate are under intensive research worldwide [83]. Disease risk ascertainment of enrollees is critical in studying efficacy of preventive interventions in randomized clinical trials [84]. An accurate predictive model can ensure enrollment of children at risk and facilitate re-analysis of earlier trials for more accurate estimates of efficacy.

Fifth, risk stratification through application of a predictive model can help clinicians and researchers properly weigh benefits against harms, costs, and inconvenience of preventive interventions for asthma [71, 85].

To facilitate asthma diagnosis and prevention, researchers have developed multiple models for predicting asthma development in children. In this paper, we provide a systematic review of these models. We present the existing models’ strengths, limitations, knowledge gaps, and opportunities for improvement in modeling. We discuss specific responses to selected gaps and limitations with the hope to stimulate future research on this topic. A list of acronyms used in this paper is provided at the end of this paper.

## Methods

This study follows the principles of the Preferred Reporting Items for Systematic Reviews and Meta-Analyses guideline [86]. All study co-authors provided input to the study protocol’s design.

### Information sources

This systematic review of published literature on predictive models for asthma development in children is limited to the period through June 3, 2015. Eight databases were used: PubMed, EMBASE, CINAHL, Scopus, the Cochrane Library, the Association for Computing Machinery (ACM) Digital Library, IEEE Xplore, and OpenGrey. EMBASE includes proceedings of 1000 conferences each year. The ACM Digital Library and IEEE Xplore are two major computer science literature databases covering journals, magazines, newsletters, and conference proceedings. OpenGrey is a database on grey literature. All citations were imported into the EndNote X7 reference management software.

### Eligibility criteria

*Inclusion criteria*: Judgment of each retrieved reference’s relevance was based on pre-defined inclusion criteria ensuring that the article’s primary focus was on predictive models for asthma development in children including ≥2 attributes. To be considered a qualified report on a predictive model, the article must report Area Under the receiver operating characteristic Curve (AUC) summarizing sensitivity and specificity, accuracy, or ≥2 of the following four performance metrics: sensitivity, specificity, positive predictive value, and negative predictive value. Reporting only one of the latter four performance metrics is insufficient for demonstrating model performance, as the model can be tuned specifically to maximize one metric by sacrificing other metrics, e.g., through a tradeoff between sensitivity and specificity.

*Exclusion criteria*: Non-English references and conference abstracts were excluded. Unlike full-length conference papers, conference abstracts provide insufficient detail of the study for meaningful review.

### Search strategies and study selection

The search strategies were developed by GL, MJ, and two medical librarians (DS and MM) trained in systematic review searches. The search queries used in the eight databases are listed in the Additional file 1. Search results were limited to human subjects and children (birth-18 years) as outlined in the Additional file 1.

For each retrieved reference, two independent reviewers (GL and MJ) evaluated the title and abstract to determine potential relevancy. For each potentially relevant reference, the full text was evaluated to make a final inclusion decision. The final literature review included articles meeting the pre-defined inclusion criteria. GL and MJ’s independent review results achieved a strong level of agreement (kappa = 0.97). Disagreements about inclusion of individual articles were addressed by discussion among GL and MJ, and if needed a third reviewer (BS).

### Data extraction and quality assessment

Two independent reviewers (GL and MJ) extracted the following article details using a data abstraction spreadsheet: purpose for making the prediction, study population, population size, methods used for building predictive models, predictors used, and the models’ performance. These two reviewers also assessed each included article’s quality using the following eight questions adapted from the Critical Appraisal Skills Programme (CASP) clinical prediction rule checklist [87]:

*Q*_*1*_: Is the predictive model clearly defined?
*Q*_*2*_: Did the population from which the predictive model was derived include an appropriate spectrum of patients?
*Q*_*3*_: Was the predictive model validated in a different group of patients?
*Q*_*4*_: Were the predictor variables and the outcome evaluated in a blinded fashion?
*Q*_*5*_: Were the predictor variables and the outcome evaluates in the whole sample selected initially?
*Q*_*6*_: Are the statistical methods used to construct and validate the predictive model clearly described?
*Q*_*7*_: Can the performance of the predictive model be calculated?
*Q*_*8*_: Was the estimate of the predictive model’s performance precise?

The CASP clinical prediction rule checklist was designed specifically for evaluating the quality of predictive modeling studies. Any discrepancy in review assessment was resolved by discussion between GL and MJ, and if needed a third reviewer (BS).

## Results

**Fig. 1:**
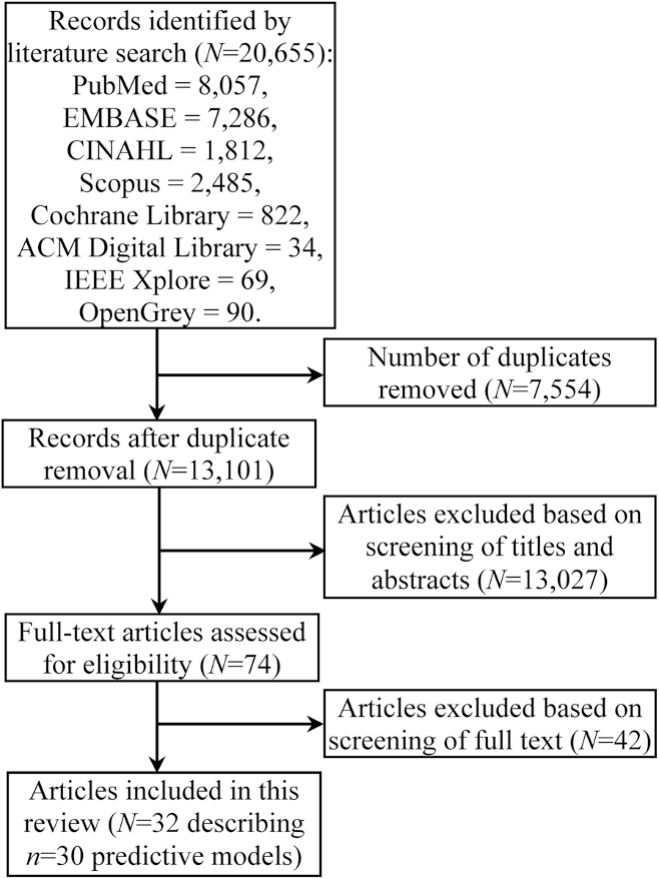
Flowchart of the article selection process

As shown in Fig. 1, the literature search returned 13,101 references in total, of which 74 were potentially relevant after review of titles and abstracts and underwent full-text review. Of those fully reviewed, 32 references describing 30 predictive models met inclusion criteria and are discussed in this paper. The other 42 were excluded because they do not primarily focus on predictive models for asthma development in children including ≥2 attributes. The included articles include only studies on predictive models. No systematic reviews or randomized controlled trials were found. In this section, we describe the state of the art of predictive models for asthma development in children. A summary of the predictive models for asthma development in children is given in Table 1. Our narrative description and the content of Table 1 are based on article details extracted into the data abstraction spreadsheet, with additional information to provide context. For the question *Q*_*3*_ used for assessing article quality, the answer is “no” for nine included articles [17, 65, 71, 88–93] and “yes” for the other 23 included articles. For each of the other seven questions *Q*_*1*_, *Q*_*2*_, and *Q*_*4*_–*Q*_*8*_ used for assessing article quality, the answer is “yes” for each included article.

**Table 1:** Categorization of existing predictive models for asthma development in children

Category	Articles (year)	Targeted population	Population size	Prediction target (the dependent variable)	Methods for building the predictive models	Predictors included in the final model	Prediction accuracy
For the general child population	Castro-Rodríguez et al. [94–96] (2000, 2011)	Children at age three	986 in [94], 1954 in [95], 93 in [96]	Asthma development at age 6–13	Clinical index	Seven predictors collected from a parental questionnaire: early wheeze, early frequent wheeze, parental asthma, eczema, blood eosinophilia, wheezing without colds, and allergic rhinitis	The loose asthma predictive index: sensitivity = 57 %, specificity = 81 %, positive predictive value = 26 %, negative predictive value = 94 % [94]
							The stringent asthma predictive index: sensitivity = 28 %, specificity = 96 %, positive predictive value = 48 %, negative predictive value = 92 % [94]
	Chang et al. [97] (2013)	Children at age 1–3	289	Asthma development at age 6–11	Clinical index	Early wheeze, early frequent wheeze, parental asthma, eczema, blood eosinophilia, wheezing without colds, allergic sensitization to aeroallergens, allergic sensitization to milk, eggs, or peanuts	Sensitivity = 17 %, specificity = 99 %, positive predictive value = 72 %, negative predictive value = 91 %
	Amat et al. [100] (2011)	Children under age three with a history of ≥3 wheezing episodes and having been assessed for respiratory wheezing disease using a standardized allergy testing program and a doctor-led ISAAC questionnaire [179, 180]	227	Asthma development at age 13			Sensitivity = 87 %, specificity = 37 %, positive predictive value = 61 %, negative predictive value = 71 %, AUC = 0.62, accuracy = 69 %
	Singer et al. [98] (2013)	Children aged 3 months–4 years with recurrent coughing or wheezing	166	Asthma development six years later	Clinical index	Early wheeze, early frequent wheeze, parental asthma, eczema, elevated fraction of exhaled nitric oxide (FeNO), wheezing without colds, allergic rhinitis	Sensitivity = 75 %, specificity = 62 %, positive predictive value = 58 %, negative predictive value = 78 %
	Amin et al. [99] (2014)	Children at age three with ≥1 parent with a positive skin prick test	589	Objectively confirmed asthma at age seven	Clinical index	Frequent wheezing, parental asthma, allergic sensitization to ≥1 aeroallergens, a history of eczema, wheezing without a cold, allergic rhinitis, allergic sensitization to milk or egg	Sensitivity = 44 %, specificity = 94 %, positive predictive value = 60 %, negative predictive value = 89 %
	Klaassen et al. [101] (2015)	Children aged 2–4 years with recurrent wheezing	198	Asthma development at age six	Logistic regression	The original asthma predictive index [94], exhaled volatile organic compounds, gene expression	AUC = 0.86
	Zhang et al. [88] (2014)	Children aged 2–20 months with the first episode of wheezing	128	Multi-trigger wheezing in the next two years	Logistic regression	Wheezing severity score computed using the Preschool Respiratory Assessment Measure scoring scale, number of shed exfoliated airway epithelial cells, family or personal history of atopic disease	Sensitivity = 95 %, specificity = 74 %, positive predictive value = 59 %, negative predictive value = 94 %
	Kurukulaaratchy et al. [89, 103] (2003, 2010)	Children at age four	1034 in [89], 936 in [103]	Persistent wheezing at age 10 (wheezing onset by age four and still wheezing at age 10)	Cumulative risk score	Four predictors collected from a parental questionnaire: family history of asthma, recurrent chest infections at age two, atopic skin prick testing at age four, and absence of nasal symptoms at age one	Sensitivity = 53 %, specificity = 85 %, positive predictive value = 68 %, negative predictive value = 74 % [89]
							Sensitivity = 22 %, specificity = 97 %, positive predictive value = 65 %, negative predictive value = 81 % [103]
	Balemans et al. [90] (2006)	Children at age two	693	Asthma development at age 21	Logistic regression	Four predictors collected from a parental questionnaire: female gender, smoking mother, lower respiratory tract illness before age two, and atopic parents	AUC = 0.66, sensitivity = 53 %, specificity = 70 %, positive predictive value = 20 %, negative predictive value = 91 %
		Children at age four				Four predictors collected from a parental questionnaire: female gender, smoking mother, lower respiratory tract illness between ages two and four, and atopic parents	AUC = 0.68, sensitivity = 71 %, specificity = 53 %, positive predictive value = 18 %, negative predictive value = 93 %
	Clough et al. [17] (1999)	Children aged 3–36 months with first wheezing in the previous 12 weeks and at least one atopic parent	97	Receiving prophylactic antiasthma treatment one year later	Logistic regression	Age, serum soluble interleukin-2 receptor (IL-2R) level	Accuracy = 71 %, sensitivity = 57 %, specificity = 84 %, positive predictive value = 76 %, negative predictive value = 68 %
	Devulapalli et al. [104] (2008)	Children at age two	449	Asthma development at age 10	Severity score	Three predictors collected from a parental questionnaire: number of episodes of bronchial obstruction, number of months with persistent bronchial obstruction, and number of hospital admissions due to bronchial obstruction	AUC = 0.78, sensitivity = 56 %, specificity = 86 %, positive predictive value = 53 %, negative predictive value = 88 % when the severity score was cut off at 5
	Marenholz et al. [105] (2009)	Infants	871	Asthma development between ages 7 and 13	Combination of two attributes	Filaggrin gene mutation, increased immunoglobulin E (IgE) levels to food allergens	Sensitivity = 9 %, specificity = 99 %, positive predictive value = 73 %, negative predictive value = 80 %
		Infants with eczema					Sensitivity = 17 %, specificity = 100 %, positive predictive value = 100 %, negative predictive value = 72 %
	Chatzimichail et al. [106–110] (2010–2013)	Children at age five with an asthma diagnosis	112	Continued asthma diagnosis at age 7–14	Evolutionary algorithm consisting of a neural network and a genetic algorithm [106]	Four predictors collected from a questionnaire: cough, bronchiolitis episodes until age five, wheezing, and asthma diagnosis [106]	Accuracy = 95 % [106]
					Principle component analysis for feature extraction, least square support vector machine for classification [107]	46 predictors collected from a questionnaire [107]	Accuracy = 96 %, sensitivity = 95 %, specificity = 96 % [107]
					Partial least square regression for feature selection, neural network for classification [108]	Nine predictors collected from a questionnaire: wheezing episodes until age five, wheezing episodes between ages three and five, wheezing episodes until age three, weight, waist’s perimeter, seasonal symptoms, FEF_25/75_, number of family members, and corticosteroids inhaled [108]	Accuracy = 97 %, sensitivity = 96 %, specificity = 100 % [108]
					Correlation analysis for feature selection, neural network for classification [109, 110]	Eight predictors collected from a questionnaire: cough, bronchiolitis episodes until age five, until age three, between ages three and five, at age two, at age three, at age four, and at age five [109]	Accuracy = 100 %, sensitivity = 100 %, specificity = 100 % [109, 110]
						Ten predictors collected from a questionnaire: cough, asthma diagnosis, total number of bronchiolitis episodes until age five, bronchiolitis episodes until age three, between ages three and five, until age four, at age one, at age two, at age three, and at age five [110]	
	Lødrup Carlsen et al. [91] (2010)	Children at birth	614	Asthma development by age 10	Logistic regression	Female gender, family network, alcohol in pregnancy, parental rhinoconjunctivis, parental education, lung function at birth (resistance ≤ median, Ve ≤ median, *t*_PTEF_/*t*_E_ ≤ 0.2)	AUC = 0.74, sensitivity = 75 %, specificity = 64 %, positive predictive value = 35 %, negative predictive value = 91 %
	Spycher et al. [92] (2012)	Children at birth	5677	Asthma development at age 7–8	Logistic regression	Genetic information	AUC < 0.6
	van der Werff et al. [102] (2013)	Children aged 4–14 without asthma	1042	Asthma development three years later	Logistic regression	Antibiotic use in the child’s first year of life, family history of atopic diseases, allergic sensitization, and municipality	AUC = 0.69
	Smolinska et al. [111] (2014)	Children aged 2–4 with recurrent wheezing symptoms	252	Asthma development at age six	Random forest for feature selection, dissimilarity partial least squares discriminant analysis for classification	Measurements of volatile organic compounds excreted in breath	Accuracy = 80 %
For the primary care setting	Vial Dupuy et al. [18] (2011)	Children under two presenting recurrent wheezing (≥3 wheezing episodes) to a pediatric pulmonology and allergy center’s outpatient department through primary care physicians’ referral	200	Development of persistent asthma at age six	Logistic regression	Family history of asthma, atopic dermatitis, multiple allergen sensitizations	AUC = 0.66, sensitivity = 42 %, specificity = 90 %, positive predictive value = 67 %, negative predictive value = 76 %
	Caudri et al. [9, 27] (2013, 2009)	Children aged 0–4 at the first time of having asthma-like symptoms in the primary care setting	2171 in [27]	Asthma development at age 7–8	Logistic regression	Eight predictors collected from a parental questionnaire: male gender, post-term delivery, parental education, parental inhaled medication, wheezing frequency, wheeze/dyspnea apart from colds, respiratory infections, and eczema	AUC = 0.74, sensitivity = 36 %, specificity = 91 %, positive predictive value = 32 %, negative predictive value = 92 %
			2877 in [9]	Asthma development at age six		Male gender, pre-term birth, parental education, parental inhaled medication, wheezing frequency, wheeze/dyspnea apart from colds, respiratory infections, eczema	AUC = 0.75, sensitivity = 37 %, specificity = 92 %, positive predictive value = 22 %, negative predictive value = 96 % when the asthma risk score corresponding to the model was cut off at 12
	van der Mark et al. [71] (2014)	Children aged 1–5 previously presented to primary care clinic for recurrent coughing, wheezing, and/or shortness of breath	438	Asthma diagnosis at age six	Logistic regression	Age, family history of asthma or allergy, wheezing-induced sleep disturbances, wheezing in the absence of common colds, specific IgE for cat, dog, and house dust mite	AUC = 0.73, positive predictive value = 22 %, negative predictive value = 78 % when the asthma prediction score corresponding to the model was cut off at 3
	Eysink et al. [65] (2005)	Children aged 1–4 who visited their primary care physicians for persistent coughing of ≥5 days	123	Asthma development at age six	Logistic regression	Age, family history of pollen allergy, wheezing, specific IgE for cat, dog, and house dust mite	AUC = 0.87
	Pescatore et al. [113, 114] (2014)	Children aged 1–3 who visited their primary care physicians for wheeze or cough	1226 in [113], 140 in [114]	Asthma development 5 years later	Logistic regression	Gender, age, wheeze without colds, wheeze frequency, activity disturbance, shortness of breath, exercise-related wheeze/cough, aeroallergen-related wheeze/cough, eczema, parental history of asthma/bronchitis	AUC = 0.76, sensitivity = 72 %, specificity = 71 %, positive predictive value = 49 %, negative predictive value = 86 % when the asthma prediction score corresponding to the model was cut off at 5
For bronchiolitis patients	Mikalsen et al. [93] (2013)	Children at age two previously hospitalized for bronchiolitis during infancy	93	Asthma diagnosis at age 11	Logistic regression	Four predictors collected from a parental questionnaire: recurrent wheezing, parental atopy, parental asthma, and atopic dermatitis	Sensitivity = 65 %, specificity = 82 %, positive predictive value ≈ 50 %, negative predictive value ≈ 89 %

### Predictive models developed for the general child population

Twenty-three models for predicting asthma development have been developed for the general child population. These models fall into the following categories: clinical index [94–100], logistic regression [17, 88, 90–92, 101, 102], cumulative risk score [89, 103], severity score [104], combination of two attributes [105], and machine learning models [106–111]. 17 models target children at or under age four [17, 88–92, 94–101, 103–105, 111]. Six of 24 studies target children with wheezing or coughing symptoms [17, 88, 98, 100, 101, 111]. Sixteen models used predictors collected from a (parental) questionnaire [89, 90, 94–98, 100–104, 106–110] and family history [88–91, 94–103, 107]. Three models used genetic information [92, 101, 105].

Different studies used differing prediction targets, candidate predictors, and populations, affecting the predictors included in the final predictive models. Age and gender were used in the predictive models in Clough et al. [17] and Balemans et al. [90, 91], respectively, but were non-predictive for the prediction target in Zhang et al. [88]. Eczema, maternal smoking, and rhinitis were used in the predictive models in Castro-Rodríguez et al. [94–100], Balemans et al. [90], and Castro-Rodríguez et al. [94–96, 98, 99], respectively, but had no independent significance for the prediction target in Kurukulaaratchy et al. [89]. Food allergy was used in the predictive models in Chang et al. [97, 99, 100, 105], but did not highly correlate with the prediction target in Kurukulaaratchy et al. [89].

Castro-Rodríguez et al. [94] published in 2000 one of the first work on predictive modeling for asthma development in children, where two clinical indices were built: the loose asthma predictive index and the stringent asthma predictive index. Both asthma predictive indices have since been externally validated, with results comparable to those of the initial study [95, 96, 98]. In addition, both asthma predictive indices have since been updated by several researchers: (1) Guilbert et al. [12] in 2004 updated the stringent asthma predictive index through replacing the predictor of allergic rhinitis by allergic sensitization to aeroallergens and allergic sensitization to milk, eggs, or peanuts [97]. (2) Singer et al. [98] in 2013 updated the original asthma predictive index through replacing the predictor of blood eosinophilia by elevated fraction of exhaled nitric oxide (FeNO) to avoid invasive blood sampling. (3) Amin et al. [99] in 2014 updated the original asthma predictive index by using the predictors of frequent wheezing, parental asthma, allergic sensitization to ≥1 aeroallergens, a history of eczema, wheezing without a cold, allergic rhinitis, and allergic sensitization to milk or egg.

Most models (17 of 23) for predicting asthma development for the general child population have low accuracy, typically with a sensitivity, positive predictive value, or AUC much less than 80 %. There are several exceptions, all with unknown performance for the situation of interest to this review:

1. The model built by Klaassen et al. [101] achieved an AUC of 0.86. In the study, prevalence of future asthma development was adjusted in the validation set through stratified sampling. It is unclear how the model would perform in the general child population, where the prevalence of future asthma development remains unmodified.
2. Chatzimichail et al. performed five studies and built one machine learning model per study [106–110]. Each study used many candidate predictors and built a model achieving an accuracy ≥95 %. Each study excluded patients with missing data representing 24 % of all patients, incurring a large selection bias. The five models predict persisting asthma in children already diagnosed. In this review, we are interested in models predicting asthma development in children who have not received an asthma diagnosis. The five studies illustrate the potential benefits of including multiple attributes and using machine learning methods in building models.

Besides the above exceptions, there are two other studies that built models with unknown performance for the situation of interest to this review. First, for children at age two, Devulapalli et al. [104] in 2008 conducted a case–control study and developed a severity score to predict asthma development at age 10. The study matched children with recurrent bronchial obstruction (≥2 episodes) to children without bronchial obstruction. Since having recurrent bronchial obstruction increases a child’s asthma risk, the matching process greatly inflated the prevalence of future asthma development in the study population. It is unclear how the model would perform in the general child population, where the matching process is absent.

Second, for children aged 6–24 months with ≥3 episodes of physician-diagnosed wheezing treated with bronchodilators or corticosteroids, Elliott et al. [112] in 2013 used single-breath FeNO >30 parts per billion (p.p.b.) to predict persistence of wheezing at age three. The prediction method achieved an AUC of 0.86, a low sensitivity of 77 %, a specificity of 94 %, a positive predictive value of 95 %, and a low negative predictive value of 73 %. Obtaining single-breath FeNO measurements requires sedating the child, special equipment, and special technical expertise. It is unknown how feasible the method will be in predicting asthma development in children. The study used a highly selected population that may have a high pre-test probability of continued wheezing at age three.

### Predictive models developed for the primary care setting

Six models for predicting asthma development in children have been developed for the primary care setting. In this setting, we prefer predictors that are accurate and non-invasive, easy, and inexpensive to obtain [90].

Among all models for predicting asthma development in children in the primary care setting, two [27, 113] have been externally validated [96, 114] with results comparable to those of the initial studies. With one exception [71], all models target children at or under age four. Three of six models used predictors collected from a parental questionnaire [9, 27, 71]. All models are based on logistic regression, target children with asthma-like symptoms such as wheezing, and used family history information. No model used genetic information.

Different studies used differing prediction targets, candidate predictors, and populations, affecting the predictors included in the final predictive models. Age was used in the predictive models in Eysink et al. [65, 71, 113, 114], but was non-predictive for the prediction target in Vial Dupuy et al. [18]. Gender was used in the predictive models in Caudri et al. [9, 27, 113, 114], but was non-predictive for the prediction targets in Vial Dupuy et al. [18, 71]. Parental asthma was used in the predictive models in Vial Dupuy et al. [18, 71, 113, 114], but had no independent significance for the prediction target in Caudri et al. [9, 27]. Parental education was used in the predictive model in Caudri et al. [9, 27], but was not collected in the studies in Vial Dupuy et al. [18, 65, 71, 113, 114].

For children aged 0–4 at the first time of having asthma-like symptoms in the primary care setting, Caudri et al. [27] in 2009 built a logistic regression model to predict asthma development at age 7–8. The model achieved a low AUC of 0.74, a low sensitivity of 36 %, a specificity of 91 %, a low positive predictive value of 32 %, and a negative predictive value of 92 %. In comparison, in the year when asthma-like symptoms were first reported, a physician’s diagnosis of asthma had a low sensitivity of 29 %, a specificity of 88 %, a low positive predictive value of 23 %, and a negative predictive value of 91 %. Thus, the model performed better than a physician’s diagnosis of asthma.

Most models (five of six) for predicting asthma development in children in the primary care setting have low accuracy, typically with an AUC much less than 80 %. There is only one exception with unknown performance for the situation of interest to this review. The model built by Eysink et al. [65] achieved an AUC of 0.87 using a case–control design matching IgE-positive children to IgE-negative children. The matching process excluded most children as they were IgE-negative. Since being IgE-positive increases a child’s asthma risk, the matching process greatly inflated the prevalence of future asthma development in the study population. It is unclear how the model would perform in routine clinical practice, where the matching process is absent. On a typical, clinically relevant child population in primary care, we would expect the model built in Eysink et al. [65] to perform worse than that built in van der Mark et al. [71], because the predictors used in the former are roughly a subset of those used in the latter while both models were developed using the same statistical method. The model built in van der Mark et al. [71] achieved a low AUC of 0.73.

### Predictive models developed for bronchiolitis patients

Asthma is highly associated with bronchiolitis, a disease primarily of children under age two. Bronchiolitis is inflammation of bronchioles, the smallest air passages in the lungs. In cases of asthma between ages 4 and 5.5, 31 % are heralded by clinically significant bronchiolitis during infancy that incurred an outpatient clinic visit, emergency department visit, and/or hospitalization [115]. By age two, >1/3 of children have experienced clinically significant bronchiolitis [116]. Between 14 and 40 % will eventually be diagnosed with asthma [117, 118], with the risk persisting into adulthood [117, 119–125]. In general, experiencing clinically significant bronchiolitis increases a child’s asthma risk 2–10 times [115, 117, 119–126].

For bronchiolitis patients, various predictors of recurrent wheezing and emerging asthma have been identified in the research literature [39, 69, 84, 119, 124, 127–154]: male gender, race, type of virus causing bronchiolitis, atopic dermatitis, family history of asthma, parental atopy, repeated wheezing at ages 0–1 and 1–2, early sensitization to common food and inhalation allergens, elevated blood eosinophils (blood eosinophilia), low serum vitamin D level, birth length, high birth weight, high weight gain from birth to hospitalization for bronchiolitis, serum eosinophil-derived neurotoxin level at 3 months after hospitalization for respiratory syncytial virus (RSV) bronchiolitis, high maternally derived RSV neutralizing antibody level in cord blood, breastfeeding <3 months, moisture in the home environment, exposure to secondhand smoke, no daycare attendance, exposure to high levels of dog allergen, swimming in chlorinated pools before age two, and the following factors during (RSV) bronchiolitis: elevated IgE values, quantity of RSV-specific IgE produced, high serum eosinophil cationic protein concentration, nasal eosinophil, high CCL5 (previously known as RANTES) level in nasal epithelia, signs of airflow limitation, monocyte interleukin-10 (IL-10) level, creola bodies in the sputum, and low serum level of soluble CD14.

For children at age two previously hospitalized for bronchiolitis during infancy, Mikalsen et al. [93] in 2013 built a logistic regression model to predict asthma diagnosis at age 11. Four predictors collected from a parental questionnaire were used: recurrent wheezing, parental atopy, parental asthma, and atopic dermatitis. The model achieved a low sensitivity of 65 %, a specificity of 82 %, a low positive predictive value around 50 %, and a negative predictive value around 89 %.

## Discussion

Existing predictive models for asthma development in children have several limitations. We now describe these limitations and identify several opportunities to improve predictive models for asthma development in children.

### Using clinical data

Most existing predictive models for asthma development in children were developed using medical research data collected specifically for the study, typically through a parental questionnaire [71]. Medical research data represent an ideal scenario atypical in practice, as they are much more robust (complete, consistent) than clinical data routinely collected in the electronic medical record in clinical practice. Also, medical research data often include additional variables not routinely collected in clinical practice. To be useful in routine clinical practice, a predictive model for asthma development in children should be developed using clinical data rather than medical research data. Such a model is suitable for implementation in an electronic medical record as a decision support tool.

### Making prediction at the right time

Most existing predictive models for asthma development in children make predictions at a time unsuitable for making clinical impact. This reduces these models’ clinical value.

Usually, a physician can use a predictive model for asthma development to facilitate asthma diagnosis and/or prevention only if the child comes to seek medical attention [27]. A patient healthcare visit, ideally for asthma-like symptoms [18], is the best time for the physician to prescribe preventive interventions for asthma and to schedule follow-up visits. However, most existing predictive models for asthma development make predictions outside of a patient healthcare visit, often when the children are at a fixed age [27]. Also, if a child is not having asthma-like symptoms at that time, it would be difficult to motivate the child and his/her parents to comply with preventive interventions and follow-up visits [55]. So far, none of the existing predictive models for asthma development in children works for all types of patient healthcare visits (outpatient clinic visit, emergency department visit, and hospitalization).

Most existing predictive models for asthma development in children were developed for relatively old children, with a median age between two and four. This age is too late for effective application of preventive interventions for asthma. Many preventive interventions are intended to modify the natural course of asthma, particularly to prevent airway remodeling and eosinophilic inflammation. Airway remodeling and eosinophilic inflammation have not occurred in children with asthma-like symptoms before age two, but are already present in asthmatic children at age two [37, 155].

To be useful in routine clinical practice, a predictive model for asthma development in children should make predictions during patient healthcare visits (possibly for asthma-like symptoms) and before children reach age two. Ideally, the model should work for all types of patient healthcare visits. In general, children at high risk for asthma should be identified as early as possible [10, 17, 18, 156]. However, this does not mean that every preventive or treatment intervention for asthma should be started immediately when a child is first predicted to be at high risk for asthma. Yoshihara [37] suggested that starting inhaled corticosteroids before age one is possibly too early and likely to have no effect on the natural history of asthma. Instead, early intervention with anti-inflammatory medications such as inhaled corticosteroids should possibly occur between ages one and three.

### Improving prediction accuracy

As mentioned in the introduction, predictive models for asthma development in children are developed to facilitate asthma diagnosis and prevention. Asthma is a non-communicable disease occurring in a minority of children. Medications that can potentially prevent asthma have side effects [55]. It is costly and unethical to give such a medication to a large proportion of children, particularly young children, for asthma prevention if they will not benefit from the medication [40, 55, 156]. The case for other interventions for asthma prevention or treatment is similar.

To be clinically valuable, a predictive model for asthma development in children needs to have both high positive predictive value and high sensitivity [157]. High positive predictive value ensures that a child with high predicted risk is indeed likely to develop asthma. High sensitivity ensures that the model can identify most children who will develop asthma in the future.

As reviewed in Results section, every existing predictive model for asthma development in children has a low AUC, a low sensitivity, and/or a low positive predictive value, typically all much less than 80 %. At present, no such model can attain accuracy high enough for routine clinical use [39, 71, 84]. It remains an open problem to improve the accuracy of predicting asthma development in children. There are several potential approaches for improving accuracy, including machine learning methods, using large data sets and exhaustive variable sets, and focusing on a child population with a high prevalence of future asthma development. We now describe these approaches individually.

#### Using machine learning methods

Most existing predictive models for asthma development in children are based on the statistical method of logistic regression. Except for those described in Chatzimichail et al. [106–111], the other existing predictive models are based on either risk score or combination of risk factors. As is the case with predictive modeling in general, machine learning methods such as support vector machines and random forests often achieve higher prediction accuracy than risk score, combination of risk factors, and logistic regression [158]. It would be interesting to compare various machine learning methods for predicting asthma development in children. Traditionally, risk score, combination of risk factors, and logistic regression have two advantages over machine learning models: easier to use and easier to interpret [159]. Through integration into a decision support tool, machine learning models can be made easy to use. Recently, a new method was developed to automatically explain the prediction results of any machine learning model without losing prediction accuracy [160]. After overcoming the barriers of difficulty in use and model interpretability, machine learning models would have no major disadvantages compared to risk score, combination of risk factors, and logistic regression.

#### Using large data sets and exhaustive variable sets

With rare exceptions [9, 27, 92], existing predictive models for asthma development in children were developed using small data sets including (typically much) fewer than 2000 children. In general, a predictive model’s accuracy improves as the training data set becomes larger, particularly if the model uses many predictors. By using data of more children to train the predictive models for asthma development in children, we are likely to improve the predictive models’ accuracy.

Many risk factors for asthma development are known in the literature [94, 161–169]. However, with few exceptions [91, 102, 106–111], most existing predictive models for asthma development in children use ≤10 attributes. By using an exhaustive set of variables coupled with a large number of children, we are likely to further improve the predictive models’ accuracy.

#### Focusing on a child population with a high prevalence of future asthma development

The positive predictive value of a model for predicting development of a disease depends critically on the prevalence of future development of the disease. The model’s positive predictive value improves as the prevalence increases [170, 171]. If the prevalence is low, which is the case for asthma in the general population, the model’s positive predictive value will not be close to 1 even if the model has both high sensitivity and high specificity [171]. This is easy to understand. In the general child population, most children are not prone to develop asthma in the future. Thus, the signal for future asthma development is weak and difficult to detect.

To address this issue and improve the predictive model’s positive predictive value, we can focus on a subset of children with a high prevalence of future asthma development rather than apply the model to the general child population [170]. The subset of children experiencing clinically significant bronchiolitis is one good such subset for several reasons. First, as mentioned at the beginning of Predictive models developed for bronchiolitis patients section, this subset of children not only has a high prevalence of future asthma development, but also includes a significant portion of children who will eventually develop asthma. Second, in this subset of children, attributes related to clinically significant bronchiolitis can provide additional information to help improve the prediction accuracy. Third, bronchiolitis mainly occurs before age two. As explained in Making prediction at the right time section, a healthcare visit for bronchiolitis is a good time to predict a child’s risk of developing asthma in the future.

So far, only one model has been developed for predicting which bronchiolitis patients will develop asthma in the future [93]. This model focuses on children at age two previously hospitalized for bronchiolitis during infancy and has two major shortcomings. First, the prediction is made at the time the child is at age two and outside of patient healthcare visit. As explained in Making prediction at the right time section, this is not a good time to make prediction. Second, among all children experiencing clinically significant bronchiolitis, only ~10 % (3 % of the general child population) are hospitalized for bronchiolitis [115, 116]. Hence, the model can identify only a small portion of children who will eventually develop asthma [115]. The narrow applicability limits the model’s usefulness.

To overcome these two shortcomings, it would be desirable to develop models for children experiencing clinically significant bronchiolitis and predict, during patient healthcare visits for bronchiolitis, which patients will develop asthma in the future. Among all children with clinically significant bronchiolitis, a subgroup analysis based on the type of healthcare visit (outpatient clinic visit, emergency department visit, and hospitalization) could evaluate how models perform on different subgroups of children. In this case, the subgroup of bronchiolitis patients in the emergency department observation unit can be either handled separately or combined into the subgroup of hospitalized patients [115].

As mentioned in Luo et al. [172], to build such predictive models, we should use risk factors for asthma development known in the literature [94, 161–169] rather than only those for bronchiolitis patients. These risk factors include both patient characteristics and environmental factors [173]. As one predictive model does not fit all [103], we should develop separate predictive models for children presenting with bronchiolitis at <6, 6–12, and 13–24 months of age [174]. As boys and girls have different likelihood of developing asthma, it could be desirable to develop separate predictive models for different genders [175].

### Using an appropriate definition of asthma

Different predictive models for asthma development in children used differing asthma definitions and predicted asthma development by various ages. This diversity impacts estimated asthma prevalence rates and the models’ prediction results [176]. At present, there is no consensus on the optimal asthma definition or age cutoff [157].

For developing a predictive model for asthma development in children, we would advocate starting from a conservative asthma definition ensuring the existence of asthma with high likelihood. One such definition is used in Schatz et al. [177]: a patient is considered to have asthma if he/she has (1) at least one ICD-9 diagnosis code of asthma (493.xx) or (2) two or more asthma-related medication dispensing (excluding oral steroids) in a 1-year period, including β-agonists (excluding oral terbutaline), inhaled steroids, other inhaled anti-inflammatory drugs, and oral leukotriene modifiers. Using a conservative asthma definition helps identify the predictors of true asthma and estimate the risk for true asthma. Then if necessary, we can broaden the scope of this definition in various ways and see how the predictive model performs with different definitions.

A child who will ever develop asthma can benefit from both timely asthma diagnosis and preventive interventions for asthma, even if he/she may outgrow his/her asthma later in life [60, 178]. Hence, we would advocate the prediction target (i.e., the dependent variable) to be ever development of asthma by a certain age rather than active asthma at a certain age. To help select an appropriate cut off age for asthma development, we can plot the cumulative rate of ever development of asthma vs. age [8, 16, 167]. The age at which the cumulative rate of ever development of asthma starts to level off can be an appropriate cut off point, as it ensures including most children who will ever develop asthma.

### Main findings

Substantial effort has been invested in predictive models for asthma development in children. Although considerable progress has been made, much remains to be done for these models to be useful in clinical practice. We have identified several limitations and open problems in predictive modeling for asthma development in children. In particular, prediction accuracy is inadequate. We have provided some preliminary thoughts on how to address these limitations and open problems. This establishes a foundation for future research on this topic.

So far, no study has deployed a predictive model for asthma development in children in clinical practice and demonstrated the model’s impact on clinicians’ behavior and clinical outcome [157]. It would be desirable to develop an accurate predictive model and then deploy it in clinical practice to measure its clinical impact, beginning at a single institution and later expanding to multiple institutions. This is essential for ensuring the model’s generalizability and for the model to be widely accepted by clinicians.

### Limitations

This systematic review has several limitations. First, by excluding articles not written in English, we may have missed predictive models for asthma development in children published in other languages. Second, there may be other predictive models for asthma development in children that have never been published and hence are missed in this systematic review. Third, few studies directly compare predictive models on the same child population. Performance metrics such as the AUC should not be used to directly compare predictive models across different child populations. Fourth, there is no clear gold standard for the prediction target of asthma development in children. Even if the approach described in Using an appropriate definition of asthma section is used to define the prediction target, the resulting definition would still be imperfect. For instance, no existing method can tell exactly which children under five have asthma, as asthma is a subjective, clinical diagnosis in this age group [14, 64, 65]. Without a gold standard definition of asthma development, it is difficult to compare the performance of different predictive models. Thus, investigation and consensus on the appropriate definition of asthma development is needed for future efforts on developing new predictive models to be clinically and widely meaningful.

## Conclusions

We systematically reviewed the literature on predictive models for asthma development in children. Existing models have several limitations. In particular, prediction accuracy is inadequate for clinical use of any existing model. Future studies will need to address these limitations to achieve optimal predictive models. More specifically, to be useful in routine clinical practice, a good predictive model should use clinical data, make prediction at a time suitable for making clinical impact, have high accuracy, and use an appropriate definition of asthma.

## Additional file

Additional file 1: **Search queries used in the eight databases.** (DOCX 87 kb)

## Abbreviations

ACM: Association for Computing Machinery
AUC: Area under the receiver operating characteristic curve
CASP: Critical Appraisal Skills Programme
FeNO: Fraction of exhaled nitric oxide
IgE: Immunoglobulin E
RSV: Respiratory syncytial virus

## References

[1] Roemer M (2011). Health care expenditures for the five most common children’s conditions, 2008: estimates for U.S. civilian noninstitutionalized children, ages 0–17. MEPS statistical brief #349.

[2] Malveaux FJ (2009). The state of childhood asthma: introduction. Pediatrics.

[3] Akinbami LJ, Moorman JE, Liu X (2011). Asthma prevalence, health care use, and mortality: United States, 2005–2009. Natl Health Stat Report.

[4] Akinbami LJ, Moorman JE, Bailey C, Zahran HS, King M, Johnson CA (2012). Trends in asthma prevalence, health care use, and mortality in the United States, 2001–2010. NCHS Data Brief.

[5] Vargas PA, Simpson PM, Bushmiaer M, Goel R, Jones CA, Magee JS (2006). Symptom profile and asthma control in school-aged children. Ann Allergy Asthma Immunol.

[6] Weissman JS, Gatsonis C, Epstein AM (1992). Rates of avoidable hospitalization by insurance status in Massachusetts and Maryland. JAMA.

[7] Wang LY, Zhong Y, Wheeler L (2005). Direct and indirect costs of asthma in school-age children. Prev Chronic Dis.

[8] Yunginger JW, Reed CE, O’Connell EJ, Melton LJ, O’Fallon WM, Silverstein MD (1992). A community-based study of the epidemiology of asthma. Incidence rates, 1964–1983. Am Rev Respir Dis.

[9] Hafkamp-de Groen E, Lingsma HF, Caudri D, Levie D, Wijga A, Koppelman GH (2013). Predicting asthma in preschool children with asthma-like symptoms: validating and updating the PIAMA risk score. J Allergy Clin Immunol.

[10] Martinez FD, Wright AL, Taussig LM, Holberg CJ, Halonen M, Morgan WJ (1995). Asthma and wheezing in the first six years of life. The Group Health Medical Associates. N Engl J Med.

[11] Martinez FD (2002). Development of wheezing disorders and asthma in preschool children. Pediatrics.

[12] Guilbert TW, Morgan WJ, Krawiec M, Lemanske RF, Sorkness C, Szefler SJ (2004). The Prevention of Early Asthma in Kids study: design, rationale and methods for the Childhood Asthma Research and Education network. Control Clin Trials.

[13] Martinez FD (2002). What have we learned from the Tucson Children’s Respiratory Study?. Paediatr Respir Rev.

[14] van de Kant KD, Klaassen EM, Jöbsis Q, Nijhuis AJ, van Schayck OC, Dompeling E (2009). Early diagnosis of asthma in young children by using non-invasive biomarkers of airway inflammation and early lung function measurements: study protocol of a case-control study. BMC Public Health.

[15] Kurukulaaratchy RJ, Fenn MH, Waterhouse LM, Matthews SM, Holgate ST, Arshad SH (2003). Characterization of wheezing phenotypes in the first 10 years of life. Clin Exp Allergy.

[16] Taussig LM, Wright AL, Holberg CJ, Halonen M, Morgan WJ, Martinez FD (2003). Tucson children’s respiratory study: 1980 to present. J Allergy Clin Immunol.

[17] Clough JB, Keeping KA, Edwards LC, Freeman WM, Warner JA, Warner JO (1999). Can we predict which wheezy infants will continue to wheeze?. Am J Respir Crit Care Med.

[18] Vial Dupuy A, Amat F, Pereira B, Labbe A, Just J (2011). A simple tool to identify infants at high risk of mild to severe childhood asthma: the persistent asthma predictive score. J Asthma.

[19] Nolte H, Nepper-Christensen S, Backer V (2006). Unawareness and undertreatment of asthma and allergic rhinitis in a general population. Respir Med.

[20] Yeatts K, Davis KJ, Sotir M, Herget C, Shy C (2003). Who gets diagnosed with asthma? Frequent wheeze among adolescents with and without a diagnosis of asthma. Pediatrics.

[21] Speight AN, Lee DA, Hey EN (1983). Underdiagnosis and undertreatment of asthma in childhood. Br Med J (Clin Res Ed).

[22] Speight AN (1978). Is childhood asthma being underdiagnosed and undertreated?. Br Med J.

[23] Majak P, Bak-Walczak E, Stelmach I, Jerzyn’ska J, Krakowiak J, Stelmach W (2011). An increasing trend of the delay in asthma diagnosis after the discontinuation of a population-based intervention. J Asthma.

[24] Pedersen S (1997). Early use of inhaled steroids in children with asthma. Clin Exp Allergy.

[25] National Asthma Education and Prevention Program. Expert panel report 3: guidelines for the diagnosis and management of asthma. 2007. Available at http://www.nhlbi.nih.gov/files/docs/guidelines/asthgdln.pdf. Accessed 10 June 2015.

[26] Lucas AE, Smeenk FW, Smeele IJ, van Schayck CP (2008). Overtreatment with inhaled corticosteroids and diagnostic problems in primary care patients, an exploratory study. Fam Pract.

[27] Caudri D, Wijga A, Schipper CM A, Hoekstra M, Postma DS, Koppelman GH (2009). Predicting the long-term prognosis of children with symptoms suggestive of asthma at preschool age. J Allergy Clin Immunol.

[28] Karadag B, Karakoc F, Ersu R, Dagli E (2007). Is childhood asthma still underdiagnosed and undertreated in Istanbul?. Pediatr Int.

[29] Lynch BA, Van Norman CA, Jacobson RM, Weaver AL, Juhn YJ (2010). Impact of delay in asthma diagnosis on health care service use. Allergy Asthma Proc.

[30] Lynch BA, Fenta Y, Jacobson RM, Li X, Juhn YJ (2012). Impact of delay in asthma diagnosis on chest X-ray and antibiotic utilization by clinicians. J Asthma.

[31] Haahtela T (1999). Early treatment of asthma. Allergy.

[32] Molis WE, Bagniewski S, Weaver AL, Jacobson RM, Juhn YJ (2008). Timeliness of diagnosis of asthma in children and its predictors. Allergy.

[33] Jones A, Sykes A (1990). The effect of symptom presentation on delay in asthma diagnosis in children in a general practice. Respir Med.

[34] Charlton I, Jones K, Bain J (1991). Delay in diagnosis of childhood asthma and its influence on respiratory consultation rates. Arch Dis Child.

[35] Anderson HR, Bailey PA, Cooper JS, Palmer JC (1981). Influence of morbidity, illness label, and social, family, and health service factors on drug treatment of childhood asthma. Lancet.

[36] Wright AL, Stern DA, Kauffmann F, Martinez FD (2006). Factors influencing gender differences in the diagnosis and treatment of asthma in childhood: the Tucson Children’s Respiratory Study. Pediatr Pulmonol.

[37] Yoshihara S (2010). Early intervention for infantile and childhood asthma. Expert Rev Clin Immunol.

[38] Murrin S (2014). CMS needs to do more to improve Medicaid children’s utilization of preventive screening services.

[39] Frey U, von Mutius E (2009). The challenge of managing wheezing in infants. N Engl J Med.

[40] Guilbert TW, Morgan WJ, Zeiger RS, Mauger DT, Boehmer SJ, Szefler SJ (2006). Long-term inhaled corticosteroids in preschool children at high risk for asthma. N Engl J Med.

[41] Nielsen KG, Bisgaard H (2000). The effect of inhaled budesonide on symptoms, lung function, and cold air and methacholine responsiveness in 2- to 5-year-old asthmatic children. Am J Respir Crit Care Med.

[42] Busse WW, Pedersen S, Pauwels RA, Tan WC, Chen YZ, Lamm CJ (2008). The Inhaled Steroid Treatment As Regular Therapy in Early Asthma (START) study 5-year follow-up: effectiveness of early intervention with budesonide in mild persistent asthma. J Allergy Clin Immunol.

[43] Haahtela T, Tamminen K, Kava T, Malmberg LP, Rytilä P, Nikander K (2009). Thirteen-year follow-up of early intervention with an inhaled corticosteroid in patients with asthma. J Allergy Clin Immunol.

[44] Pauwels RA, Pedersen S, Busse WW, Tan WC, Chen YZ, Ohlsson SV (2003). Early intervention with budesonide in mild persistent asthma: a randomised, double-blind trial. Lancet.

[45] Morgan WJ, Crain EF, Gruchalla RS, O’Connor GT, Kattan M, Evans R (2004). Results of a home-based environmental intervention among urban children with asthma. N Engl J Med.

[46] The Childhood Asthma Management Program Research Group (2000). Long-term effects of budesonide or nedocromil in children with asthma. N Engl J Med.

[47] Chen YZ, Busse WW, Pedersen S, Tan W, Lamm CJ, O’Byrne PM (2006). Early intervention of recent onset mild persistent asthma in children aged under 11 yrs: the Steroid Treatment As Regular Therapy in early asthma (START) trial. Pediatr Allergy Immunol.

[48] Selroos O, Löfroos AB, Pietinalho A, Riska H (2004). Asthma control and steroid doses 5 years after early or delayed introduction of inhaled corticosteroids in asthma: a real-life study. Respir Med.

[49] Adams RJ, Fuhlbrigge A, Finkelstein JA, Lozano P, Livingston JM, Weiss KB (2001). Impact of inhaled antiinflammatory therapy on hospitalization and emergency department visits for children with asthma. Pediatrics.

[50] Haahtela T, Tuomisto LE, Pietinalho A, Klaukka T, Erhola M, Kaila M (2006). A 10 year asthma programme in Finland: major change for the better. Thorax.

[51] O’Byrne PM, Barnes PJ, Rodriguez-Roisin R, Runnerstrom E, Sandstrom T, Svensson K (2001). Low dose inhaled budesonide and formoterol in mild persistent asthma: the OPTIMA randomized trial. Am J Respir Crit Care Med.

[52] Agertoft L, Pedersen S (1994). Effects of long-term treatment with an inhaled corticosteroid on growth and pulmonary function in asthmatic children. Respir Med.

[53] Weiss K, Buxton M, Andersson FL, Lamm CJ, Liljas B, Sullivan SD (2006). Cost-effectiveness of early intervention with once-daily budesonide in children with mild persistent asthma: results from the START study. Pediatr Allergy Immunol.

[54] van Wonderen KE, van der Mark LB, Mohrs J, Geskus RB, van der Wal WM, van Aalderen WM (2009). Prediction and treatment of asthma in preschool children at risk: study design and baseline data of a prospective cohort study in general practice (ARCADE). BMC Pulm Med.

[55] König P (1997). Evidence for benefits of early intervention with non-steroidal drugs in asthma. Pediatr Pulmonol Suppl.

[56] O’Byrne PM, Pedersen S, Busse WW, Tan WC, Chen YZ, Ohlsson SV (2006). Effects of early intervention with inhaled budesonide on lung function in newly diagnosed asthma. Chest.

[57] O’Byrne PM, Pedersen S, Lamm CJ, Tan WC, Busse WW (2009). Severe exacerbations and decline in lung function in asthma. Am J Respir Crit Care Med.

[58] Selroos O, Pietinalho A, Löfroos AB, Riska H (1995). Effect of early vs late intervention with inhaled corticosteroids in asthma. Chest.

[59] Suissa S, Ernst P, Benayoun S, Baltzan M, Cai B (2000). Low-dose inhaled corticosteroids and the prevention of death from asthma. N Engl J Med.

[60] Panhuysen CI, Vonk JM, Koëter GH, Schouten JP, van Altena R, Bleecker ER (1997). Adult patients may outgrow their asthma: a 25-year follow-up study. Am J Respir Crit Care Med.

[61] Clark NM, Brown R, Joseph CL, Anderson EW, Liu M, Valerio M (2002). Issues in identifying asthma and estimating prevalence in an urban school population. J Clin Epidemiol.

[62] Galant SP, Crawford LJ, Morphew T, Jones CA, Bassin S (2004). Predictive value of a cross-cultural asthma case-detection tool in an elementary school population. Pediatrics.

[63] Robertson CF, Rubinfeld AR, Bowes G (1992). Pediatric asthma deaths in Victoria: the mild are at risk. Pediatr Pulmonol.

[64] Pedersen S (2007). Preschool asthma–not so easy to diagnose. Prim Care Respir J.

[65] Eysink PE, ter Riet G, Aalberse RC, van Aalderen WM, Roos CM, van der Zee JS (2005). Accuracy of specific IgE in the prediction of asthma: development of a scoring formula for general practice. Br J Gen Pract.

[66] Humbert M (2006). The right tools at the right time. Chest.

[67] Roberts G (2009). Predicting the long-term outcome of preschool wheeze: are we there yet?. J Allergy Clin Immunol.

[68] Bush A (2007). Diagnosis of asthma in children under five. Prim Care Respir J.

[69] Shinohara M, Wakiguchi H, Saito H, Matsumoto K (2008). Presence of eosinophils in nasal secretion during acute respiratory tract infection in young children predicts subsequent wheezing within two months. Allergol Int.

[70] Wever-Hess J, Kouwenberg JM, Duiverman EJ, Hermans J, Wever AM (1999). Prognostic characteristics of asthma diagnosis in early childhood in clinical practice. Acta Paediatr.

[71] van der Mark LB, van Wonderen KE, Mohrs J, van Aalderen WM, ter Riet G, Bindels PJ (2014). Predicting asthma in preschool children at high risk presenting in primary care: development of a clinical asthma prediction score. Prim Care Respir J.

[72] Sly PD, Boner AL, Björksten B, Bush A, Custovic A, Eigenmann PA (2008). Early identification of atopy in the prediction of persistent asthma in children. Lancet.

[73] Yoshihara S, Ono M, Yamada Y, Fukuda H, Abe T, Arisaka O (2009). Early intervention with suplatast tosilate for prophylaxis of pediatric atopic asthma: a pilot study. Pediatr Allergy Immunol.

[74] Warner JO (2001). A double-blinded, randomized, placebo-controlled trial of cetirizine in preventing the onset of asthma in children with atopic dermatitis: 18 months’ treatment and 18 months’ posttreatment follow-up. J Allergy Clin Immunol.

[75] Lukkarinen M, Lukkarinen H, Lehtinen P, Vuorinen T, Ruuskanen O, Jartti T (2013). Prednisolone reduces recurrent wheezing after first rhinovirus wheeze: a 7-year follow-up. Pediatr Allergy Immunol.

[76] Szefler SJ (2014). Advances in pediatric asthma in 2013: coordinating asthma care. J Allergy Clin Immunol.

[77] Hatakka K, Savilahti E, Pönkä A, Meurman JH, Poussa T, Näse L (2001). Effect of long term consumption of probiotic milk on infections in children attending day care centres: double blind, randomised trial. BMJ.

[78] Custovic A, Simpson BM, Simpson A, Kissen P, Woodcock A (2001). Effect of environmental manipulation in pregnancy and early life on respiratory symptoms and atopy during first year of life: a randomised trial. Lancet.

[79] Niggemann B, Jacobsen L, Dreborg S, Ferdousi HA, Halken S, Høst A (2006). Five-year follow-up on the PAT study: specific immunotherapy and long-term prevention of asthma in children. Allergy.

[80] Jacobsen L, Niggemann B, Dreborg S, Ferdousi HA, Halken S, Høst A (2007). Specific immunotherapy has long-term preventive effect of seasonal and perennial asthma: 10-year follow-up on the PAT study. Allergy.

[81] Möller C, Dreborg S, Ferdousi HA, Halken S, Høst A, Jacobsen L (2002). Pollen immunotherapy reduces the development of asthma in children with seasonal rhinoconjunctivitis (the PAT-study). J Allergy Clin Immunol.

[82] Becker A, Watson W, Ferguson A, Dimich-Ward H, Chan-Yeung M (2004). The Canadian asthma primary prevention study: outcomes at 2 years of age. J Allergy Clin Immunol.

[83] Holt PG, Sly PD (2007). Prevention of allergic respiratory disease in infants: current aspects and future perspectives. Curr Opin Allergy Clin Immunol.

[84] Mansbach JM, Camargo CA (2009). Respiratory viruses in bronchiolitis and their link to recurrent wheezing and asthma. Clin Lab Med.

[85] Global strategy for asthma management and prevention. Global Initiative for Asthma (GINA) 2014. Available at http://www.ginasthma.org/documents/4, 2014. Accessed 10 June 2015.

[86] Moher D, Liberati A, Tetzlaff J, Altman DG, The PRISMA Group (2009). Preferred Reporting Items for Systematic Reviews and Meta-Analyses: the PRISMA statement. PLoS Med.

[87] Critical Appraisal Skills Programme (CASP) clinical prediction rule checklist. http://media.wix.com/ugd/dded87_9f84310697164809ac7392ab63f3d8ca.pdf, 2015. Accessed 10 June 2015.

[88] Zhang Y, Zhou C, Liu J, Yang H, Zhao S (2014). A new index to identify risk of multi-trigger wheezing in infants with first episode of wheezing. J Asthma.

[89] Kurukulaaratchy RJ, Matthews S, Holgate ST, Arshad SH (2003). Predicting persistent disease among children who wheeze during early life. Eur Respir J.

[90] Balemans WA, van der Ent CK, Schilder AG, Sanders EA, Zielhuis GA, Rovers MM (2006). Prediction of asthma in young adults using childhood characteristics: Development of a prediction rule. J Clin Epidemiol.

[91] Lødrup Carlsen KC, Mowinckel P, Granum B, Carlsen KH (2010). Can childhood asthma be predicted at birth?. Clin Exp Allergy.

[92] Spycher BD, Henderson J, Granell R, Evans DM, Smith GD, Timpson NJ (2012). Genome-wide prediction of childhood asthma and related phenotypes in a longitudinal birth cohort. J Allergy Clin Immunol.

[93] Mikalsen IB, Halvorsen T, Eide GE, Øymar K (2013). Severe bronchiolitis in infancy: can asthma in adolescence be predicted?. Pediatr Pulmonol.

[94] Castro-Rodríguez JA, Holberg CJ, Wright AL, Martinez FD (2000). A clinical index to define risk of asthma in young children with recurrent wheezing. Am J Respir Crit Care Med.

[95] Leonardi NA, Spycher BD, Strippoli MP, Frey U, Silverman M, Kuehni CE (2011). Validation of the Asthma Predictive Index and comparison with simpler clinical prediction rules. J Allergy Clin Immunol.

[96] Rodriguez-Martinez CE, Sossa-Briceño MP, Castro-Rodriguez JA (2011). Discriminative properties of two predictive indices for asthma diagnosis in a sample of preschoolers with recurrent wheezing. Pediatr Pulmonol.

[97] Chang TS, Lemanske RF, Guilbert TW, Gern JE, Coen MH, Evans MD (2013). Evaluation of the modified asthma predictive index in high-risk preschool children. J Allergy Clin Immunol Pract.

[98] Singer F, Luchsinger I, Inci D, Knauer N, Latzin P, Wildhaber JH (2013). Exhaled nitric oxide in symptomatic children at preschool age predicts later asthma. Allergy.

[99] Amin P, Levin L, Epstein T, Ryan P, LeMasters G, Khurana Hershey G (2014). Optimum predictors of childhood asthma: persistent wheeze or the Asthma Predictive Index?. J Allergy Clin Immunol Pract.

[100] Amat F, Vial A, Pereira B, Petit I, Labbe A, Just J (2011). Predicting the long-term course of asthma in wheezing infants is still a challenge. ISRN Allergy.

[101] Klaassen EM, van de Kant KD, Jöbsis Q, van Schayck OC, Smolinska A, Dallinga JW (2015). Exhaled biomarkers and gene expression at preschool age improve asthma prediction at 6 years of age. Am J Respir Crit Care Med.

[102] van der Werff SD, Junco Díaz R, Reyneveld R, Heymans MW, Ponce Campos M, Gorbea Bonet M (2013). Prediction of asthma by common risk factors: a follow-up study in Cuban schoolchildren. J Investig Allergol Clin Immunol.

[103] Matricardi PM, Illi S, Keil T, Wagner P, Wahn U, Lau S (2010). Predicting persistence of wheezing: one algorithm does not fit all. Eur Respir J.

[104] Devulapalli CS, Carlsen KC, Håland G, Munthe-Kaas MC, Pettersen M, Mowinckel P (2008). Severity of obstructive airways disease by age 2 years predicts asthma at 10 years of age. Thorax.

[105] Marenholz I, Kerscher T, Bauerfeind A, Esparza-Gordillo J, Nickel R, Keil T (2009). An interaction between filaggrin mutations and early food sensitization improves the prediction of childhood asthma. J Allergy Clin Immunol.

[106] Chatzimichail E, Paraskakis E, Rigas A (2013). An evolutionary two-objective genetic algorithm for asthma prediction. Proceedings of UKSim’13.

[107] Chatzimichail E, Paraskakis E, Sitzimi M, Rigas A (2013). An intelligent system approach for asthma prediction in symptomatic preschool children. Comput Math Methods Med.

[108] Chatzimichail E, Paraskakis E, Rigas A (2013). Predicting asthma outcome using partial least square regression and artificial neural networks. Adv Artificial Intelligence.

[109] Chatzimichail E, Paraskakis E, Sitzimi M, Rigas A (2011). Predicting the long-term outcome of preschool children with asthma symptoms. Proceedings of EHB’11.

[110] Chatzimichail EA, Rigas AG, Paraskakis EN (2010). An artificial intelligence technique for the prediction of persistent asthma in children. Proceedings of ITAB’10.

[111] Smolinska A, Klaassen EM, Dallinga JW, van de Kant KD, Jobsis Q, Moonen EJ (2014). Profiling of volatile organic compounds in exhaled breath as a strategy to find early predictive signatures of asthma in children. PLoS One.

[112] Elliott M, Heltshe SL, Stamey DC, Cochrane ES, Redding GJ, Debley JS (2013). Exhaled nitric oxide predicts persistence of wheezing, exacerbations, and decline in lung function in wheezy infants and toddlers. Clin Exp Allergy.

[113] Pescatore AM, Dogaru CM, Duembgen L, Silverman M, Gaillard EA, Spycher BD (2014). A simple asthma prediction tool for preschool children with wheeze or cough. J Allergy Clin Immunol.

[114] Grabenhenrich LB, Reich A, Fischer F, Zepp F, Forster J, Schuster A (2014). The novel 10-item asthma prediction tool: external validation in the German MAS birth cohort. PLoS One.

[115] Carroll KN, Wu P, Gebretsadik T, Griffin MR, Dupont WD, Mitchel EF (2009). The severity-dependent relationship of infant bronchiolitis on the risk and morbidity of early childhood asthma. J Allergy Clin Immunol.

[116] Zorc JJ, Hall CB (2010). Bronchiolitis: recent evidence on diagnosis and management. Pediatrics.

[117] Piippo-Savolainen E, Korppi M (2008). Wheezy babies-wheezy adults? Review on long-term outcome until adulthood after early childhood wheezing. Acta Paediatr.

[118] Perlstein PH, Kotagal UR, Bolling C, Steele R, Schoettker PJ, Atherton HD (1999). Evaluation of an evidence-based guideline for bronchiolitis. Pediatrics.

[119] Hyvärinen M, Piippo-Savolainen E, Korhonen K, Korppi M (2005). Teenage asthma after severe infantile bronchiolitis or pneumonia. Acta Paediatr.

[120] Larouch V, Rivard G, Deschesnes F, Goulet R, Turcotte H, Boulet LP (2000). Asthma and airway hyper-responsiveness in adults who required hospital admission for bronchiolitis in early childhood. Respir Med.

[121] McConnochie KM, Roghmann KJ (1984). Bronchiolitis as a possible cause of wheezing in childhood: new evidence. Pediatrics.

[122] Piippo-Savolainen E, Remes S, Kannisto S, Korhonen K, Korppi M (2004). Asthma and lung function 20 years after wheezing in infancy: results from a prospective follow-up study. Arch Pediatr Adolesc Med.

[123] Sigurs N, Bjarnason R, Sigurbergsson F, Kjellman B (2000). Respiratory syncytial virus bronchiolitis in infancy is an important risk factor for asthma and allergy at age 7. Am J Respir Crit Care Med.

[124] Singh AM, Moore PE, Gern JE, Lemanske RF, Hartert TV (2007). Bronchiolitis to asthma: a review and call for studies of gene-virus interactions in asthma causation. Am J Respir Crit Care Med.

[125] Ruotsalainen M, Piippo-Savolainen E, Hyvärinen MK, Korppi M (2010). Adulthood asthma after wheezing in infancy: a questionnaire study at 27 years of age. Allergy.

[126] James KM, Gebretsadik T, Escobar GJ, Wu P, Carroll KN, Li SX (2013). Risk of childhood asthma following infant bronchiolitis during the respiratory syncytial virus season. J Allergy Clin Immunol.

[127] Moore HC, de Klerk N, Holt P, Richmond PC, Lehmann D (2012). Hospitalisation for bronchiolitis in infants is more common after elective caesarean delivery. Arch Dis Child.

[128] Stensballe LG, Ravn H, Kristensen K, Agerskov K, Meakins T, Aaby P (2009). Respiratory syncytial virus neutralizing antibodies in cord blood, respiratory syncytial virus hospitalization, and recurrent wheeze. J Allergy Clin Immunol.

[129] Khaldi E, Joulak M, Jawahdou F (1999). Infant asthma in Tunisia. Pediatr Pulmonol Suppl.

[130] Bont L, Heijnen CJ, Kavelaars A, van Aalderen WM, Brus F, Draaisma JT (2000). Monocyte IL-10 production during respiratory syncytial virus bronchiolitis is associated with recurrent wheezing in a one-year follow-up study. Am J Respir Crit Care Med.

[131] Bont L, Van Aalderen WM, Versteegh J, Brus F, Draaisma JT, Pekelharing-Berghuis M (2001). Airflow limitation during respiratory syncytial virus lower respiratory tract infection predicts recurrent wheezing. Pediatr Infect Dis J.

[132] Chung HL, Kim SG (2002). RANTES may be predictive of later recurrent wheezing after respiratory syncytial virus bronchiolitis in infants. Ann Allergy Asthma Immunol.

[133] Kotaniemi-Syrjänen A, Reijonen TM, Korhonen K, Korppi M (2002). Wheezing requiring hospitalization in early childhood: predictive factors for asthma in a six-year follow-up. Pediatr Allergy Immunol.

[134] Pifferi M, Ragazzo V, Caramella D, Baldini G (2001). Eosinophil cationic protein in infants with respiratory syncytial virus bronchiolitis: predictive value for subsequent development of persistent wheezing. Pediatr Pulmonol.

[135] Piippo-Savolainen E, Remes S, Kannisto S, Korhonen K, Korppi M (2006). Early predictors for adult asthma and lung function abnormalities in infants hospitalized for bronchiolitis: a prospective 18- to 20-year follow-up. Allergy Asthma Proc.

[136] Piippo-Savolainen E, Remes S, Korppi M (2007). Does early exposure or sensitization to inhalant allergens predict asthma in wheezing infants? A 20-year follow-up. Allergy Asthma Proc.

[137] Piippo-Savolainen E, Remes S, Korppi M (2007). Does blood eosinophilia in wheezing infants predict later asthma? A prospective 18-20-year follow-up. Allergy Asthma Proc.

[138] Reijonen TM, Korppi M, Kuikka L, Savolainen K, Kleemola M, Mononen I (1997). Serum eosinophil cationic protein as a predictor of wheezing after bronchiolitis. Pediatr Pulmonol.

[139] Soferman R, Bar-Zohar D, Jurgenson U, Fireman E (2004). Soluble CD14 as a predictor of subsequent development of recurrent wheezing in hospitalized young children with respiratory syncytial virus-induced bronchiolitis. Ann Allergy Asthma Immunol.

[140] Schuurhof A, Janssen R, de Groot H, Hodemaekers HM, de Klerk A, Kimpen JL (2011). Local interleukin-10 production during respiratory syncytial virus bronchiolitis is associated with post-bronchiolitis wheeze. Respir Res.

[141] Welliver RC, Sun M, Rinaldo D, Ogra PL (1986). Predictive value of respiratory syncytial virus-specific IgE responses for recurrent wheezing following bronchiolitis. J Pediatr.

[142] Yamada Y, Yoshihara S (2010). Creola bodies in infancy with respiratory syncytial virus bronchiolitis predict the development of asthma. Allergol Int.

[143] Ruotsalainen M, Hyvärinen MK, Piippo-Savolainen E, Korppi M (2013). Adolescent asthma after rhinovirus and respiratory syncytial virus bronchiolitis. Pediatr Pulmonol.

[144] Ehlenfield DR, Cameron K, Welliver RC (2000). Eosinophilia at the time of respiratory syncytial virus bronchiolitis predicts childhood reactive airway disease. Pediatrics.

[145] Cassimos DC, Tsalkidis A, Tripsianis GA, Stogiannidou A, Anthracopoulos M, Ktenidou-Kartali S (2008). Asthma, lung function and sensitization in school children with a history of bronchiolitis. Pediatr Int.

[146] Bacharier LB, Cohen R, Schweiger T, Yin-Declue H, Christie C, Zheng J (2012). Determinants of asthma after severe respiratory syncytial virus bronchiolitis. J Allergy Clin Immunol.

[147] Beigelman A, Bacharier LB (2013). The role of early life viral bronchiolitis in the inception of asthma. Curr Opin Allergy Clin Immunol.

[148] Escobar GJ, Ragins A, Li SX, Prager L, Masaquel AS, Kipnis P (2010). Recurrent wheezing in the third year of life among children born at 32 weeks’ gestation or later: relationship to laboratory-confirmed, medically attended infection with respiratory syncytial virus during the first year of life. Arch Pediatr Adolesc Med.

[149] Kuikka L, Reijonen T, Remes K, Korppi M (1994). Bronchial asthma after early childhood wheezing: a follow-up until 4.5-6 years of age. Acta Paediatr.

[150] Midulla F, Pierangeli A, Cangiano G, Bonci E, Salvadei S, Scagnolari C (2012). Rhinovirus bronchiolitis and recurrent wheezing: 1-year follow-up. Eur Respir J.

[151] Nuolivirta K, Koponen P, Helminen M, Korppi M (2012). Weight gain in infancy and post-bronchiolitis wheezing. Acta Paediatr.

[152] Koponen P, Helminen M, Paassilta M, Luukkaala T, Korppi M (2012). Preschool asthma after bronchiolitis in infancy. Eur Respir J.

[153] Kim CK, Seo JK, Ban SH, Fujisawa T, Kim DW, Callaway Z (2013). Eosinophil-derived neurotoxin levels at 3 months post-respiratory syncytial virus bronchiolitis are a predictive biomarker of recurrent wheezing. Biomarkers.

[154] Valkonen H, Waris M, Ruohola A, Ruuskanen O, Heikkinen T (2009). Recurrent wheezing after respiratory syncytial virus or non-respiratory syncytial virus bronchiolitis in infancy: a 3-year follow-up. Allergy.

[155] Saglani S, Payne DN, Zhu J, Wang Z, Nicholson AG, Bush A (2007). Early detection of airway wall remodeling and eosinophilic inflammation in preschool wheezers. Am J Respir Crit Care Med.

[156] Martinez FD (1999). Recognizing early asthma. Allergy.

[157] Savenije OE, Kerkhof M, Koppelman GH, Postma DS (2012). Predicting who will have asthma at school age among preschool children. J Allergy Clin Immunol.

[158] Steyerberg EW (2009). Clinical prediction models: a practical approach to development, validation, and updating.

[159] Freitas AA (2013). Comprehensible classification models: a position paper. SIGKDD Explorations.

[160] Luo G, Stone BL, Sakaguchi F, Sheng X, Murtaugh MA. Using computational approaches to improve risk-stratified patient management: rationale and methods. JMIR Res Protoc. 2015;4(4):e128. 10.2196/resprot.5039 PMC4704915 26503357

[161] Oliveti JF, Kercsmar CM, Redline S (1996). Pre- and perinatal risk factors for asthma in inner city African-American children. Am J Epidemiol.

[162] Arruda LK, Solé D, Baena-Cagnani CE, Naspitz CK (2005). Risk factors for asthma and atopy. Curr Opin Allergy Clin Immunol.

[163] Sherman CB, Tosteson TD, Tager IB, Speizer FE, Weiss ST (1990). Early childhood predictors of asthma. Am J Epidemiol.

[164] Voisin C, Sardella A, Marcucci F, Bernard A (2010). Infant swimming in chlorinated pools and the risks of bronchiolitis, asthma and allergy. Eur Respir J.

[165] Karunasekera KA, Jayasinghe JA, Alwis LW (2001). Risk factors of childhood asthma: a Sri Lankan study. J Trop Pediatr.

[166] Litonjua AA, Weiss ST (2015). Risk factors for asthma.

[167] Matricardi PM, Illi S, Grüber C, Keil T, Nickel R, Wahn U (2008). Wheezing in childhood: incidence, longitudinal patterns and factors predicting persistence. Eur Respir J.

[168] Belsky DW, Sears MR (2014). The potential to predict the course of childhood asthma. Expert Rev Respir Med.

[169] Bannier MA, van de Kant KD, Jöbsis Q, Dompeling E (2015). Biomarkers to predict asthma in wheezing preschool children. Clin Exp Allergy.

[170] Akobeng AK (2007). Understanding diagnostic tests 1: sensitivity, specificity and predictive values. Acta Paediatr.

[171] Altman DG, Bland JM (1994). Diagnostic tests 2: Predictive values. BMJ.

[172] Luo G, Nkoy FL, Gesteland PH, Glasgow TS, Stone BL (2014). A systematic review of predictive modeling for bronchiolitis. Int J Med Inform.

[173] The International Study of Asthma and Allergies in Childhood (ISAAC) Steering Committee (1998). Worldwide variation in prevalence of symptoms of asthma, allergic rhinoconjunctivitis, and atopic eczema: ISAAC. Lancet.

[174] Korppi M (2010). Asthma predictive factors in infants with bronchiolitis: asthma risk at 13-20 years of age. Eur Respir J.

[175] Wen HJ, Chiang TL, Lin SJ, Guo YL (2015). Predicting risk for childhood asthma by pre-pregnancy, perinatal, and postnatal factors. Pediatr Allergy Immunol.

[176] Van Wonderen KE, Van Der Mark LB, Mohrs J, Bindels PJ, Van Aalderen WM, Ter Riet G (2010). Different definitions in childhood asthma: how dependable is the dependent variable?. Eur Respir J.

[177] Schatz M, Cook EF, Joshua A, Petitti D (2003). Risk factors for asthma hospitalizations in a managed care organization: development of a clinical prediction rule. Am J Manag Care.

[178] Andersson M, Hedman L, Bjerg A, Forsberg B, Lundbäck B, Rönmark E (2013). Remission and persistence of asthma followed from 7 to 19 years of age. Pediatrics.

[179] Bacharier LB, Boner A, Carlsen KH, Eigenmann PA, Frischer T, Götz M (2008). Diagnosis and treatment of asthma in childhood: a PRACTALL consensus report. Allergy.

[180] Asher MI, Montefort S, Björkstén B, Lai CK, Strachan DP, Weiland SK (2006). Worldwide time trends in the prevalence of symptoms of asthma, allergic rhinoconjunctivitis, and eczema in childhood: ISAAC Phases One and Three repeat multicountry cross-sectional surveys. Lancet.

