# Supplementary material for: A systematic review of predictive models for asthma development in children
Source: BMC Med Inform Decis Mak. 2015 Nov 28;15:99. doi: 10.1186/s12911-015-0224-9 (PMC4662818; doi:10.1186/s12911-015-0224-9)
Supplement: Additional file 1: — Search queries used in the eight databases. (DOCX 87 kb) [file 12911_2015_224_MOESM1_ESM.docx]

**Additional file 1. Search queries used in the eight databases**

1. Pubmed

("asthma"[MeSH Terms] OR asthma[tw] OR asthmatic[tw] OR asthmatics[tw] OR wheeze[tw] OR wheezing[tw] OR "bronchial hyperreactivity"[Mesh] OR bronchial hyperreactivity[tw])

AND

(infant[tw] OR infants[tw] OR infancy[tw] OR newborn[tw] OR newborns[tw] OR neonate[tw] OR neonates[tw] OR neonatal[tw] OR toddler[tw] OR toddlers[tw] OR child[tw] OR children[tw] OR childrens[tw] OR childhood[tw] OR adolescent[tw] OR adolescents[tw] OR adolescence[tw] OR teenage[tw] OR teenager[tw] OR teenagers[tw] OR teen[tw] OR teens[tw])

AND

("Models, Statistical"[MeSH Terms] OR "Logistic Models"[Mesh] OR "Algorithms"[MeSh] OR model[tiab] OR models[tiab] OR modeling[tiab] OR tool[tw] OR tools[tw] OR index[tw] OR indices[tw] OR questionnaire*[tw] OR score[tw] OR scores[tw] OR regression analysis[tw] OR multivariate analysis[tw] OR validation[tw] OR validating[tw] OR validated[tw] OR rule[tiab] OR rules[tiab] OR predictive signature*[tw] OR interaction[ti] OR algorithm*[tw])

AND

("Risk"[Mesh] OR "Forecasting"[Mesh] OR predict*[tw] OR probability[tw] OR likelihood[tw] OR projection[tw] OR projections[tw] OR forecast*[tw] OR odds ratio*[tw] OR incidence[tw] OR prevalence[tw] OR risk[tw])

AND

("Diagnosis"[MeSH Terms] OR "Diagnosis, Differential"[Mesh] OR "diagnosis"[subheading] OR "Prognosis"[MeSH Terms] OR diagnostic[tw] OR prognosis[tw] OR prognostic[tw] OR assessment[tw] OR assessments[tw] OR clinical feature*[tw] OR prevent*[tw] OR prophylactic*[tw] OR prophylax*[tw] OR diagnosis[tw] OR criteria[tw] OR evaluation[tiab])

NOT

("Animals"[Mesh] NOT "Humans"[Mesh])

1. EMBASE

('asthma'/exp OR 'wheezing'/exp OR asthma:ab,ti OR asthmatic:ab,ti OR wheeze:ab,ti OR wheezing:ab,ti OR 'bronchial hyperreactivity':ab,ti)

AND

('juvenile'/exp OR infant:ab,ti OR infants:ab,ti OR infancy:ab,ti OR newborn:ab,ti OR newborns:ab,ti OR neonate:ab,ti OR neonates:ab,ti OR neonatal:ab,ti OR toddler:ab,ti OR toddlers:ab,ti OR child:ab,ti OR children:ab,ti OR childrens:ab,ti OR childhood:ab,ti OR adolescent:ab,ti OR adolescents:ab,ti OR adolescence:ab,ti OR teenage:ab,ti OR teenager:ab,ti OR teenagers:ab,ti OR teen:ab,ti OR teens:ab,ti OR juvenile:ab,ti OR juveniles:ab,ti)

AND

('statistical model'/exp OR 'logistic models' OR 'questionnaire'/exp OR 'multivariate analysis'/exp OR 'regression analysis'/exp OR 'algorithm'/exp OR 'delphi study'/exp OR 'validation study'/exp OR model:ab,ti OR models:ab,ti OR modelling:ab,ti OR tool:ab,ti OR tools:ab,ti OR index:ab,ti OR indices:ab,ti OR questionnaire:ab,ti OR questionnaires:ab,ti OR score:ab,ti OR scores:ab,ti OR 'regression analysis':ab,ti OR 'multivariate analysis':ab,ti OR validation:ab,ti OR validating:ab,ti OR validated:ab,ti OR rule:ab,ti OR rules:ab,ti OR (predictive NEXT/1 (signature? OR interaction OR algorithm?)):ab,ti OR (delphi NEXT/2 (technique? OR study OR method? OR process OR approach)):ab,ti)

AND

('risk'/exp OR 'prediction and forecasting'/exp OR 'maximum likelihood method'/exp OR 'probability'/exp OR 'incidence'/exp OR 'prevalence'/de OR risk:ab,ti OR forecasting:ab,ti OR prediction:ab,ti OR predict:ab,ti OR predicts:ab,ti OR predicted:ab,ti OR probability:ab,ti OR likelihood:ab,ti OR projection:ab,ti OR prevalence:ab,ti)

AND

('diagnosis'/exp OR 'differential diagnosis'/exp OR 'prognosis'/de OR 'prevention'/exp OR 'prophylaxis'/exp OR 'disease course'/exp OR 'clinical feature'/exp OR 'symptomatology'/exp OR diagnosis:ab,ti OR diagnostic:ab,ti OR prognosis:ab,ti OR prognostic:ab,ti OR assessment:ab,ti OR assessments:ab,ti OR 'clinical feature':ab,ti OR 'clinical features':ab,ti OR prevent:ab,ti OR prevents:ab,ti OR prevented:ab,ti OR prevention:ab,ti OR prophylactic:ab,ti OR prophylactics:ab,ti OR prophylaxis:ab,ti OR criteria:ab,ti OR evaluation:ab,ti OR 'disease course':ab,ti OR 'disease development':ab,ti OR 'disease marker':ab,ti)

NOT

('animal'/exp NOT 'human'/exp)

1. CINAHL

(MH "Asthma+" OR TX "asthma" OR TX "asthmatic*" OR TX "wheez*" OR TX "bronchial hyperreactivity")

AND

(TX "infant" OR TX "infants" OR TX "infancy" OR TX "newborn*" OR TX "neonat*" OR TX "toddler*" OR "child*" OR TX "adolescent" OR TX "adolescenc*" OR TX "teen*")

AND

(MH "Models, Statistical" OR MH "Predictive Value of Tests" OR MH "Questionnaires+" OR MH "Algorithms" OR TX "logistic model*" OR TX "logic model*" OR TX "model" OR TX "models" OR TX "modeling" OR TX "modelling" OR TX "regression" OR TX "statistic*" OR TX "index" OR TX "indexes" OR TX "indices" OR TX "score" OR TX "scoring" OR TX "scores" OR TX "questionnaire*" OR TX "survey" OR TX "surveys" OR TX "predictive signature*" OR TX "algorithm*" OR TX "validation" OR TX "validating" OR TX "validated" OR TX "rule" OR TX "rules" OR TX "interaction*")

AND

(TW "risk" OR TW "risks" OR MH "forecasting" OR TW "forecast*" OR TX "predict*" OR TX "probability" OR TX "projection" OR TX "projections" OR TX "odds ratio*" OR TX "incidence" OR TX "prevalence" OR TW "likelihood")

AND

(MH "Diagnosis+" OR MH "Diagnosis, Differential" OR MH "Prognosis+" OR TW "prognosis" OR TX "prognostic*" OR TX "diagnostic" OR TX "prophylactic*" OR TX "prophylax*" OR TX "assessment*" OR TX "prevent*" OR TX "clinical feature*" OR TX "confirmed" OR TX "evaluation" OR TX "evaluations" OR TX "criteria")

NOT

(MH "Vertebrates+" NOT (MH "Human"))

1. Scopus

TITLE-ABS-KEY(asthma OR asthmatic OR asthmatics OR wheeze OR wheezing OR "bronchial hyperreactivity")

AND

TITLE-ABS-KEY(infant OR infants OR infancy OR newborn OR newborns OR neonate OR neonates OR neonatal OR toddler OR toddlers OR child OR children OR childrens OR children's OR childhood OR adolescent OR adolescents OR adolescence OR teenage OR teenager OR teenagers OR teen OR teens)

AND

(TITLE-ABS-KEY((statistical W/2 model*) OR (logistic W/2 model*) OR ((regression OR multivariate) W/2 analysis) OR (model OR models OR modeling OR tool OR tools OR index OR indices OR questionnaire* OR score OR scores OR validation OR validating OR validated OR rule OR rules OR predictive signature* OR algorithm*)) OR (TITLE(interaction)))

AND

TITLE-ABS-KEY(risk OR forecast* OR predict* OR probability OR likelihood OR projection OR projections OR (odds W/2 ratio*) OR incidence OR prevalence)

AND

TITLE-ABS-KEY(diagnosis OR diagnostic OR prognosis OR prognostic OR assessment OR assessments OR (clinical PRE/1 feature*) OR "disease course" OR prevent* OR prophylactic* OR prophylax* OR criteria OR evaluation)

AND NOT

TITLE-ABS-KEY((animal OR animals) AND NOT (human OR humans))

1. The Cochrane Library

(asthma:ti,ab,kw (Word variations have been searched) OR MeSH descriptor: [Asthma] explode all trees OR wheez*:ti,ab,kw OR MeSH descriptor: [Bronchial Hyperreactivity] explode all trees)

AND

(infant:ti,ab,kw (Word variations have been searched) OR newborn:ti,ab,kw (Word variations have been searched) OR neonatal:ti,ab,kw (Word variations have been searched) OR toddler:ti,ab,kw (Word variations have been searched) OR child:ti,ab,kw (Word variations have been searched) OR adolescent:ti,ab,kw (Word variations have been searched) OR teen:ti,ab,kw (Word variations have been searched) OR teenage:ti,ab,kw (Word variations have been searched) OR teenager:ti,ab,kw (Word variations have been searched))

AND

(MeSH descriptor: [Models, Statistical] explode all trees OR MeSH descriptor: [Logistic Models] explode all trees OR MeSH descriptor: [Algorithms] explode all trees OR model:ti,ab,kw (Word variations have been searched) OR modeling:ti,ab,kw (Word variations have been searched) OR tool:ti,ab,kw (Word variations have been searched) OR index:ti,ab,kw (Word variations have been searched) OR questionnaire:ti,ab,kw (Word variations have been searched) OR score:ti,ab,kw (Word variations have been searched) OR regression analysis:ti,ab,kw (Word variations have been searched) OR multivariate analysis:ti,ab,kw (Word variations have been searched) OR validation:ti,ab,kw (Word variations have been searched) OR validating:ti,ab,kw (Word variations have been searched) OR validate:ti,ab,kw (Word variations have been searched) OR rule:ti,ab,kw OR rules:ti,ab,kw OR predictive signature*:ti,ab,kw OR interaction:ti,ab,kw (Word variations have been searched) OR algorithm:ti,ab,kw (Word variations have been searched))

AND

(MeSH descriptor: [Risk] explode all trees OR MeSH descriptor: [Forecasting] explode all trees OR predict*:ti,ab,kw OR probability:ti,ab,kw (Word variations have been searched) OR likelihood:ti,ab,kw (Word variations have been searched) OR projection:ti,ab,kw (Word variations have been searched) OR forecast*:ti,ab,kw OR odds ratio*:ti,ab,kw OR incidence:ti,ab,kw OR prevalence:ti,ab,kw OR risk:ti,ab,kw (Word variations have been searched))

AND

(MeSH descriptor: [Diagnosis] explode all trees OR MeSH descriptor: [Diagnosis, Differential] explode all trees OR Any MeSH descriptor with qualifier(s): [Diagnosis - DI] OR MeSH descriptor: [Prognosis] explode all trees OR diagnosis:ti,ab,kw (Word variations have been searched) OR diagnostic:ti,ab,kw (Word variations have been searched) OR prognosis:ti,ab,kw (Word variations have been searched) OR prognostic:ti,ab,kw (Word variations have been searched) OR assessment:ti,ab,kw (Word variations have been searched) OR prophylactic*:ti,ab,kw OR prophylax*:ti,ab,kw OR clinical feature*:ti,ab,kw OR prevent*:ti,ab,kw OR criteria:ti,ab,kw OR evaluation:ti,ab,kw (Word variations have been searched))

NOT

(MeSH descriptor: [Animals] explode all trees NOT MeSH descriptor: [Humans] explode all trees)

1. The ACM Digital Library

(Title:asthma OR Title:asthmatic OR Title:asthmatics OR Title:wheeze OR Title:wheezing OR Title:"bronchial hyperreactivity" OR Abstract:asthma OR Abstract:asthmatic OR Abstract:asthmatics OR Abstract:wheeze OR Abstract:wheezing OR Abstract:"bronchial hyperreactivity")

AND

(Title:risk OR Title:forecasting OR Title:forecast OR Title:forecasts OR Title:predict OR Title:predicts OR Title:predictive OR Title:prediction OR Title:predictions OR Title:probability OR Title:likelihood OR Title:projection OR Title:projections OR Title:"odds ratio" OR Title:"odds ratios" OR Title:incidence OR Title:prevalence OR Abstract:Risk OR Abstract:Forecasting OR Abstract:forecast OR Abstract:forecasts OR Abstract:predict OR Abstract:predicts OR Abstract:predictive OR Abstract:prediction OR Abstract:predictions OR Abstract:probability OR Abstract:likelihood OR Abstract:projection OR Abstract:projections OR Abstract:"odds ratio" OR Abstract:"odds ratios" OR Abstract:incidence OR Abstract:prevalence)

AND

(PublishedAs:journal OR PublishedAs:proceeding OR PublishedAs:transaction)

1. IEEE Xplore

(asthma* OR wheeze OR wheezing OR "bronchial hyperreactivity")

AND

(risk OR likelihood OR forecast* OR probability OR projection* OR odds ratio* OR incidence OR prevalence OR predict*)

1. OpenGrey

(asthma* OR wheeze OR wheezing OR “bronchial hyperreactivity”)

AND

(risk OR forecast* OR predict* OR probability OR likelihood OR projection* OR “odds ratio*” OR incidence OR prevalence)

AND

(model* OR algorithm* OR tool* OR index* OR indices OR questionnaire* OR score* OR survey* OR “predictive signature*” OR regression* OR multivariate OR validat* OR rule OR rules OR interaction*)
